# Incarceration History and Access to and Receipt of Health Care in the US

**DOI:** 10.1001/jamahealthforum.2023.5318

**Published:** 2024-02-23

**Authors:** Jingxuan Zhao, Jessica Star, Xuesong Han, Zhiyuan Zheng, Qinjin Fan, Sylvia Kewei Shi, Stacey A. Fedewa, K. Robin Yabroff, Leticia M. Nogueira

**Affiliations:** 1 Surveillance and Health Equity Science, American Cancer Society, Atlanta, Georgia; 2 Department of Hematology and Oncology, Emory University, Atlanta, Georgia

## Abstract

**Importance:**

People with a history of incarceration may experience barriers in access to and receipt of health care in the US.

**Objective:**

To examine the associations of incarceration history and access to and receipt of care and the contribution of modifiable factors (educational attainment and health insurance coverage) to these associations.

**Design, Setting, and Participants:**

Individuals with and without incarceration history were identified from the 2008 to 2018 National Longitudinal Survey of Youth 1979 cohort. Analyses were conducted from October 2022 to December 2023.

**Main Measures and Outcomes:**

Access to and receipt of health care were measured as self-reported having usual source of care and preventive service use, including physical examination, influenza shot, blood pressure check, blood cholesterol level check, blood glucose level check, dental check, and colorectal, breast, and cervical cancer screenings across multiple panels. To account for the longitudinal study design, we used the inverse probability weighting method with generalized estimating equations to evaluate associations of incarceration history and access to care. Separate multivariable models examining associations between incarceration history and receipt of each preventive service adjusted for sociodemographic factors; sequential models further adjusted for educational attainment and health insurance coverage to examine their contribution to the associations of incarceration history and access to and receipt of health care.

**Results:**

A total of 7963 adults with 41 614 person-years of observation were included in this study; of these, 586 individuals (5.4%) had been incarcerated, with 2800 person-years of observation (4.9%). Compared with people without incarceration history, people with incarceration history had lower percentages of having a usual source of care or receiving preventive services, including physical examinations (69.6% vs 74.1%), blood pressure test (85.6% vs 91.6%), blood cholesterol level test (59.5% vs 72.2%), blood glucose level test (61.4% vs 69.4%), dental check up (51.1% vs 66.0%), and breast (55.0% vs 68.2%) and colorectal cancer screening (65.6% vs 70.3%). With additional adjustment for educational attainment and health insurance, the associations of incarceration history and access to care were attenuated for most measures and remained statistically significant for measures of having a usual source of care, blood cholesterol level test, and dental check up only.

**Conclusions and Relevance:**

The results of this survey study suggest that incarceration history was associated with worse access to and receipt of health care. Educational attainment and health insurance may contribute to these associations. Efforts to improve access to education and health insurance coverage for people with an incarceration history might mitigate disparities in care.

## Key Points

**Question:**

Is incarceration history associated with access to and receipt of health care in the US?

**Findings:**

In this survey study of 7963 individuals with and without an incarceration history, people with an incarceration history were less likely to have a usual source of care and receive preventive services, such as physical examinations, influenza shots, and colorectal and breast cancer screenings.

**Meaning:**

The study results suggest that efforts to improve access to health care among people with an incarceration history are warranted to reduce public health inequities.

## Teaser

This survey study examines the associations of incarceration history and access to and receipt of care and the contribution of educational attainment and health insurance coverage to these associations.

## Introduction

The incarceration rates in the US are high, with prison and jail incarceration rates of 350 and 192 per 100 000 residents in 2021, respectively.^1,2^ Carceral systems include nearly 1600 state prisons, 98 federal prisons, more than 3000 local jails, and 1300 juvenile correctional facilities, as well as other facilities.^3^ Based on 2016 prison admission rates, an estimated 9% of male and 2% of female individuals will be incarcerated during their lifetime.^4^ Meanwhile, Black people are disproportionality incarcerated in the US,^1,2^ reflecting historic and current institutional racism within the criminal justice system, including discriminatory policing, prosecution, and sentencing practices. Defined as a key social determinant of health by Healthy People 2030, incarceration has been associated with several adverse health outcomes, including infectious diseases, chronic conditions, mental health disorders, and increased mortality.^5,6,7,8^

The physical, psychological, and socioeconomic consequences of being incarcerated may exacerbate inequities in access to health care.^5^ Compared with individuals without any history of incarceration, people with incarceration history are less likely to have health insurance coverage, which is a strong determinant of access to care.^9,10,11^ Additionally, health care workers are more likely to discriminate against individuals who report recent interactions with the judicial system, which compounds barriers to access and receipt of health care.^12^ Several studies reported that people with incarceration history had limited access to care and worse health outcomes.^9,13,14,15,16,17^ For example, a study found that less than half of women with a history of incarceration in New York, New York, reported any primary care utilization, such as visiting a physician’s office or outpatient clinics, within 1 year after release, which is lower than the national average.^17^ Several studies found that people who were currently incarcerated were less likely to receive breast and colorectal cancer screenings and also less likely to receive follow-up care after having an abnormal screening result.^18,19,20,21,22,23,24^ However, most prior studies were cross-sectional,^9,14,17^ conducted in a single city or state,^17^ or focused only on highly selected populations,^10,14,16^ such as young adults.^10,16^ One study used longitudinal data to examine the association of incarceration history and receipt of preventive services among adults aged 29 years^16^; however, multiple preventive services, such as colorectal and breast cancer screenings, are not recommended for this age. In addition, all studies examining incarceration and cancer screening were based on data from a few local jails or 1 state prison with a relatively small sample size and did not include a control group.^18,19,20,21,24^

In this study, we used national longitudinal data to examine the association of incarceration history and access to and receipt of health care. We focused on many preventive services recommended by US Preventive Services Task Force (USPSTF). In addition, we focused on people aged 43 to 62 years, an age group for which incidence of chronic conditions increases and for whom preventive services are crucial for their health. Given that 19.3% and 20.1% of people in this sample who had been incarcerated were first incarcerated between the ages of 12 to 18 years and 19 to 22 years, respectively, and incarceration at young age may contribute their life trajectory and access to education and health insurance,^9,10,11,25^ we also examined the contributions of educational attainment and health insurance on these associations between incarceration history and preventive services use.

## Methods

### Data and Sample

We used data from the National Longitudinal Survey of Youth 1979 (NLSY79), a longitudinal survey of a closed cohort of individuals who were born during 1957 through 1964 (aged 14-22 years when first interviewed in 1979).^26^ Sponsored by the US Bureau of Labor Statistics, interviews were conducted annually from 1979 to 1994 and on a biennial basis thereafter, with a retention rate of 69.0% through the entire follow-up period. We used data from round 23 (2008-2009) to round 28 (2018-2019), when information on access to and receipt of health care was systematically collected. We excluded individuals with missing information on incarceration history from the study. If a respondent reported residing at a jail, prison, or detention facility, they were ineligible for the study outcomes during that round and excluded from the sample for that round. Because deidentified NLSY79 data are publicly available, this study was exempt from institutional review board review and informed consent was waived.

### Measures

Incarceration history was a time-varying, self-reported measured generated by 2 survey questions. First, respondents who answered yes to the question about incarceration history in round 2 (1980) (“Have you ever been sentenced to spend time in a corrections institution, like a jail, prison, or a youth institution like a training school or reform school?") were identified as having an incarceration history. Second, at each subsequent interview round, respondents who reported residence in jail, prison, or other detention facilities in rounds 3 to 27 were also identified as having a history of incarceration. For example, if a respondent was initially incarcerated in round 25, this person would be categorized as not having an incarceration history in rounds 23 to 24 and with an incarceration history in rounds 26 to 28.

Having a usual source of care was self-reported and defined as having a health care clinician to see when sick or in need of health advice. Use of preventive services recommended by the USPSTF was measured with questions about receipt of a physical examination, influenza shot, blood pressure check, blood cholesterol level check, blood glucose level check, and dental check during the previous 24 months. Receipt of cancer screenings recommended by the USPSTF, including breast, colorectal, and cervical cancer screenings, was also collected at each interview round from 2008 onward. To approximate up-to-date cancer screenings, we took advantage of the longitudinal design and generated variables to measure receipt of breast cancer screenings during the past 2 years among women aged 50 to 74 years, receipt of colorectal cancer screening during the previous 2, 4, 6, 8, and 10 years among men and women aged 50 to 75 years, and receipt of cervical cancer screening during the previous 2 and 4 years among women aged 21 to 65 years who did not undergo a hysterectomy. More specifically, we included rounds 23 to 28 for receipt of health care during the previous 2 years, rounds 24 to 28 for the previous 4 years, rounds 25 to 28 for the previous 6 years, rounds 26 to 28 for the previous 8 years, and rounds 27 to 28 for the previous 10 years. Exact wording of survey questions and the eligible population for each preventive service are listed in eTable 1 in Supplement 1.

### Statistical Analysis

In this study, we used the longitudinal design with data from the NLSY79 round 23 to 28, which allowed us to account for the sequence of events (bidirectional association between incarceration history and access to and receipt of health care), measure changes over time (such as incarceration history), and include individuals with loss-to-follow-up at each interview round. Descriptive statistics were used to compare sociodemographic characteristics by incarceration history at the person-years level to reflect the longitudinal design.

The inverse probability weighting (IPW) approach was used to account for the potential bias introduced by differential loss to follow-up by incarceration history at each interview round. Because individuals with an incarceration history might be more likely to be lost to follow-up, each participant was weighted by the inverse of the probability of being incarcerated and the probability of being absent from the analysis. Because men and people with lower socioeconomic status are more likely to be incarcerated, both IPWs were conditional on time-fixed measures of sex, self-reported race and ethnicity, and parents’ highest educational attainment (a proxy for baseline socioeconomic status),^1,27^ as well as time-varying measures of age using logistic regression. With the IPW approach, individuals who were more likely to be lost to follow-up were assigned a higher weight.

Generalized estimating equations with the IPWs were used to analyze the longitudinal data; these equations produce population average estimates while accounting for within-participant associations. We used SAS, PROC GENMOD (SAS Institute), with a binomial distribution and logit link to assess the associations of incarceration history and study outcomes. The IPW was included in the analysis with the weight statement. To account for the sequence of events, time-varying variables, including incarceration history, geographic region, rurality, educational attainment, number of health conditions, and health insurance status at the time of interview (yes/no), were lagged for 1 interview round in the models. For example, when examining the association of incarceration history and receipt of colorectal cancer screening during the previous 10 years, we used incarceration history information in rounds 26 and 27 and past 10-year colorectal cancer screening information in rounds 27 and 28, respectively. Detailed information on which rounds of data were used for the analysis is shown in eTable 2 in Supplement 1.

In multivariable models, single-year age, sex, geographic region (past round), rurality (past round), parents’ highest educational attainment, number of health conditions (past round), and survey year were included as covariates. These covariates were included because they were presumed, a priori, to be possible confounders in the association between incarceration and access to and receipt of health care. We included self-reported race and ethnicity in the models as a proxy for exposure to racism, as racialized populations are more likely to be incarcerated due to institutional racism within the criminal justice system, and they also have worse access to health care.^1,2,28^ In addition, to test if educational attainment and health insurance contributed to the disparities in access to care by incarceration history, educational attainment (past round) and health insurance status (past round) were added separately and together in sequential adjustment, which is a commonly used method to examine the contribution of a factor to an association.^29,30^ We also examined the association of time since last incarceration (never incarcerated, >10 years, and 0-10 years) and access to and receipt of health care.

As suggested by the NLSY97 analytic guidance and consistent with earlier studies,^11,31,32^ sample weight and survey design were used for descriptive statistics but not for regressions. SAS, version 9.4 (SAS Institute), was used for data analysis. Statistical tests were 2 sided, and α was set at .05.

## Results

A total of 7963 adults with 41 614 person-years of observation were included in this study. Among this population, 586 individuals had been incarcerated (5.4%), with 2800 person-years of observation (4.9%). Respondents racialized as White and women were less likely to have incarceration history. Incarceration history was more common among respondents whose parents had lower educational attainment and respondents with lower educational attainment and without health insurance coverage. Among people who had been incarcerated, 180 (6.3%) were last incarcerated within the past 0 to 10 years, and 2620 (93.7%) were last incarcerated more than 10 years ago (Table 1).

**Table 1.** Sample Characteristics of Participants in the National Longitudinal Survey of Youth 1979 From 2008 to 2018

Characteristic	Ever incarcerated (past round)			
	No		Yes	
	Person-years	Weighted % (SE)	Person-years	Weighted % (SE)
Total	38 814	100	2800	100
Age, mean (SE), y	52.29 (0.03)		52.16 (0.11)	
Sex				
Female	21 405	51.47 (0.30)	347	14.25 (0.86)
Male	17 409	48.53 (0.30)	2453	85.75 (0.86)
Race and ethnicity				
Hispanic	7017	6.69 (0.10)	611	10.75 (0.55)
Non-Hispanic Black	11 294	12.86 (0.14)	1397	31.12 (0.94)
Non-Hispanic White	19 664	77.75 (0.20)	696	52.48 (1.18)
Other^a^	470	1.46 (0.08)	76	4.21 (0.54)
Missing	369	1.24 (0.07)	20	1.44 (0.33)
Region (past round)				
Northeast	5772	16.64 (0.23)	372	10.34 (0.64)
Midwest	9101	28.54 (0.28)	497	22.13 (1.04)
South	16 251	36.86 (0.29)	1255	44.62 (1.20)
West	7429	17.42 (0.23)	621	21.92 (1.02)
Missing	261	0.54 (0.04)	55	0.98 (0.14)
Rurality (past round)				
Urban	29 023	70.25 (0.28)	2283	77.20 (1.07)
Rural	8756	27.30 (0.28)	422	20.48 (1.05)
Missing	1035	2.46 (0.09)	95	2.32 (0.31)
Educational attainment (past round)				
<High school graduate	3067	5.97 (0.14)	616	19.69 (0.94)
High school graduate	15 453	38.37 (0.29)	1515	52.54 (1.21)
Some college or more	18 703	51.68 (0.30)	495	21.85 (1.07)
Missing	1591	3.97 (0.12)	174	5.91 (0.57)
No. of health conditions (past round)				
0	26 544	69.30 (0.28)	1919	66.58 (1.15)
1	9085	23.14 (0.25)	617	23.07 (1.03)
≥2	3185	7.55 (0.16)	264	10.36 (0.74)
With health insurance (past round)^b^				
Yes	33 402	88.33 (0.19)	1768	64.79 (1.15)
No	5381	11.62 (0.19)	1029	35.08 (1.14)
Educational attainment of parents (highest)				
<High school graduate	12 510	22.42 (0.24)	1286	40.40 (1.17)
High school graduate	14 540	40.79 (0.30)	968	39.37 (1.20)
Some college or more	10 524	34.62 (0.30)	360	16.03 (0.93)
Missing	1240	2.17 (0.08)	186	4.20 (0.35)
Time since last incarceration^b^				
>10 y	NA	NA	2620	93.70 (0.57)
0-10 y	NA	NA	180	6.30 (0.57)

Abbreviation: NA, not applicable.

^a^ Includes American Indian or Alaska Native, Asian, and Native Hawaiian or Other Pacific Islander.

^b^ Missing category was not displayed due to the small number.

**Figure 1.**
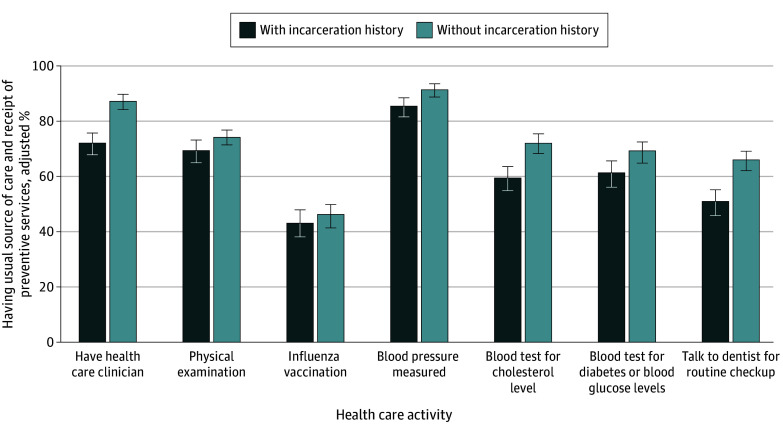
Adjusted Percentages of Having a Usual Source of Care and Receipt of Preventive Services by Incarceration History Error bars represent 95% CIs.

**Figure 2.**
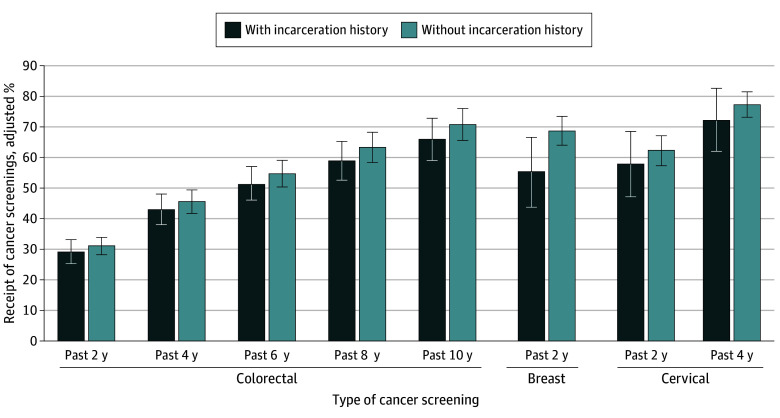
Adjusted Percentages of Receipt of Cancer Screenings by Incarceration History Error bars represent 95% CIs.

After adjusting for sociodemographic characteristics, compared with people without an incarceration history, people with an incarceration history had lower percentages of having usual source of care (72.1% vs 87.4%) and receiving preventive services, including physical examination (69.6% vs 74.2%), blood pressure test (85.6% vs 91.6%), blood cholesterol level test (59.5% vs 72.2%), blood glucose level test (61.4% vs 69.4%), dental check up (51.1% vs 66.0%), and breast cancer screening (55.0% vs 68.2%) during the previous 2 years and colorectal cancer screening (65.6% vs 70.3%) during the previous 10 years (Figure 1 and Figure 2, Table 2).

**Table 2.** Differences in Predicted Margins in Access to and Receipt of Health Care Among People With and Without an Incarceration History^a^

Characteristic	Differences in predicted margins among those with incarceration history vs without incarceration history, % (95% CI)			
	Model 1^b^	Model 2^c^	Model 3^d^	Model 4^e^
Have health care clinician to see when sick or in need of health advice	−15.30 (−18.27 to −12.33)	−12.54 (−15.48 to −9.6)	−8.16 (−10.52 to −5.79)	−6.66 (−9.02 to −4.3)
Receipt of preventive services				
Physical examination	−4.55 (−7.28 to −1.82)	−3.14 (−5.88 to −0.4)	0.48 (−2.06 to 3.01)	1.07 (−1.48 to 3.61)
Influenza shot	−3.12 (−6.64 to 0.4)	−1.21 (−4.74 to 2.33)	0.73 (−2.69 to 4.16)	1.88 (−1.56 to 5.33)
Blood pressure measured	−6.00 (−8.35 to −3.66)	−4.20 (−6.57 to −1.82)	−2.81 (−5.05 to −0.57)	−1.58 (−3.83 to 0.68)
Blood test for cholesterol level	−12.74 (−15.94 to −9.54)	−9.97 (−13.13 to −6.81)	−6.97 (−9.91 to −4.03)	−5.32 (−8.25 to −2.39)
Blood test for diabetes or blood glucose levels	−7.99 (−11.12 to −4.87)	−5.74 (−8.86 to −2.63)	−3.24 (−6.18 to −0.30)	−1.91 (−4.85 to 1.04)
Talk to dentist for routine check up	−14.96 (−18.21 to −11.71)	−10.64 (−13.9 to −7.37)	−8.52 (−11.53 to −5.50)	−5.61 (−8.66 to −2.56)
Colonoscopy or other colorectal cancer screening				
Past 2 y	−2.11 (−5.3 to 1.08)	−1.30 (−4.51 to 1.91)	1.11 (−2.01 to 4.24)	1.44 (−1.70 to 4.57)
Past 4 y	−2.68 (−6.64 to 1.28)	−1.51 (−5.48 to 2.47)	1.98 (−1.87 to 5.83)	2.31 (−1.57 to 6.18)
Past 6 y	−3.49 (−7.79 to 0.82)	−1.82 (−6.13 to 2.49)	1.25 (−2.92 to 5.41)	1.96 (−2.22 to 6.14)
Past 8 y	−4.33 (−8.86 to 0.21)	−2.55 (−7.07 to 1.97)	0.24 (−4.15 to 4.62)	1.08 (−3.31 to 5.46)
Past 10 y	−4.71 (−9.41 to −0.01)	−3.23 (−7.94 to 1.48)	−0.24 (−4.91 to 4.43)	0.41 (−4.27 to 5.08)
Mammogram or radiography for breast cancer screening				
Past 2 y	−13.24 (−23.8 to −2.69)	−11.27 (−21.56 to −0.99)	−9.50 (−18.96 to −0.05)	−8.21 (−17.51 to 1.10)
Papanicolaou test for cervical cancer screening				
Past 2 y	−4.52 (−14.48 to 5.44)	−2.65 (−12.47 to 7.17)	−1.59 (−11.28 to 8.1)	−0.34 (−9.95 to 9.26)
Past 4 y	−5.08 (−14.87 to 4.72)	−3.35 (−13.01 to 6.32)	−2.45 (−12.09 to 7.20)	−1.09 (−10.62 to 8.44)

^a^ Data are from the National Longitudinal Survey of Youth 1979, 2008 to 2018.

^b^ Models adjust for age, sex, race and ethnicity, parents’ highest educational attainment, rural/urban status (last round), region (last round), No. of conditions (last round), and survey year.

^c^ Models adjust for age, sex, race and ethnicity, parents’ highest educational attainment, rural/urban status (last round), region (last round), No. of conditions (last round), survey year, and educational attainment (last round).

^d^ Models adjust for age, sex, race and ethnicity, parents’ highest educational attainment, rural/urban status (last round), region (last round), No. of conditions (last round), survey year, and health insurance (last round).

^e^ Models adjust for age, sex, race and ethnicity, parents’ highest educational attainment, rural/urban status (last round), region (last round), No. of conditions (last round), survey year, educational attainment (last round), and health insurance (last round).

With additional adjustment for educational attainment (past round), the associations of incarceration history and access to and receipt of care were attenuated but remained statistically significant for some outcomes (Table 2; eTable 3 in Supplement 1). With additional adjustment for health insurance coverage (past round), the associations of incarceration history and access to and receipt of health care were attenuated and remained statistically significant for a few measures, including having a clinician to see when sick or in need of care, blood pressure test, blood test for cholesterol level, blood glucose level test, dental check up, and breast cancer screening (Table 2; eTable 3 in Supplement 1). After adjusting for demographic factors and educational attainment and health insurance altogether, associations of incarceration history and access to and receipt of health care were further attenuated and remained significant only for measures of having a clinician to see when sick or in need of care, blood test for cholesterol level, and dental check up (Table 2; eTable 3 in Supplement 1).

When stratifying the analysis by recency of last incarceration, compared with people who had never been incarcerated, people last incarcerated more than 10 years before had lower percentages of having a usual source of care and receiving most preventive services, and people last incarcerated within 10 years had lower percentages of having a usual source of care and receiving some preventive services. People last incarcerated within 10 years had a lower percentage of receiving breast cancer screening during the previous 2 years than people last incarcerated more than 10 years prior (Table 3).

**Table 3.** Differences in Predicted Margins in Access to and Receipt of Health Care Among People Last Incarcerated Less Than 10 Years Ago, Last Incarcerated 0 to 10 Years Ago, and Those Who Were Never Incarcerated^a^

Characteristic	Differences in predicted margins, % (95% CI)^b^		
	Last incarcerated >10 y vs without incarceration history	Last incarcerated 0-10 y vs without incarceration history	Last incarcerated 0-10 y vs last incarcerated >10 y
Have health care clinician to see when sick or in need of health advice	−15.03 (−18.09 to −11.97)	−18.04 (−27.55 to −8.53)	−3.01 (−12.79 to 6.77)
Receipt of preventive services			
Physical examination	−4.51 (−7.32 to −1.70)	−4.94 (−13.43 to 3.55)	−0.43 (−9.11 to 8.25)
Influenza shot	−3.57 (−7.17 to 0.03)	1.50 (−10.51 to 13.52)	5.08 (−7.22 to 17.37)
Blood pressure measured	−6.54 (−8.96 to −4.11)	−0.20 (−5.12 to 4.72)	6.34 (1.23 to 11.45)
Blood test for cholesterol level	−12.59 (−15.83 to −9.35)	−14.28 (−26.24 to −2.33)	−1.69 (−13.87 to 10.48)
Blood test for diabetes or blood glucose levels	−7.81 (−11.01 to −4.60)	−9.92 (−19.97 to 0.12)	−2.12 (−12.41 to 8.18)
Talk to dentist for routine check up	−14.44 (−17.75 to −11.13)	−20.30 (−30.82 to −9.77)	−5.85 (−16.6 to 4.89)
Colonoscopy or other colorectal cancer screening			
Past 2 y	−2.67 (−5.84 to 0.50)	4.45 (−11.17 to 20.07)	7.12 (−8.79 to 23.02)
Past 4 y	−3.04 (−7.02 to 0.94)	1.46 (−15.87 to 18.78)	4.50 (−13.14 to 22.14)
Past 6 y	−4.16 (−8.44 to 0.13)	4.25 (−15.28 to 23.78)	8.41 (−11.40 to 28.23)
Past 8 y	−5.58 (−10.03 to −1.14)	11.00 (−11.50 to 33.50)	16.58 (−6.15 to 39.31)
Past 10 y	−5.72 (−10.31 to −1.14)	7.21 (−15.97 to 30.39)	12.93 (−10.48 to 36.34)
Mammogram or radiography for breast cancer screening			
Past 2 y	−8.12 (−16.91 to 0.68)	−55.14 (−79.51 to −30.76)	−47.02 (−73.38 to −20.66)
Papanicolaou test for cervical cancer screening			
Past 2 y	−0.93 (−10.62 to 8.77)	−29.14 (−59.13 to 0.85)	−28.21 (−60.14 to 3.72)
Past 4 y	−2.98 (−12.71 to 6.76)	−20.95 (−51.18 to 9.28)	−17.97 (−49.94 to 14.00)

^a^ Data from National Longitudinal Survey of Youth 1979, 2008 to 2018.

^b^ Models adjusted for age, sex, race and ethnicity, parents’ highest educational attainment, rural/urban status (past round), region (past round), number of conditions (past round), and survey year.

## Discussion

Using national longitudinal data, the findings of this study suggested that incarceration history was associated with lower percentages of having a usual source of care and receipt of recommended preventive services, such as physical examinations, influenza shot, and blood pressure check. People with incarceration history were also less likely to receive colorectal and breast cancer screenings than individuals without incarceration history. We also found that educational attainment and health insurance contributed to disparities in access to care by incarceration history. Our findings suggest that incarceration history is associated with worse access to and receipt of care, which may be associated with worse health outcomes and translate into public health inequities.

Prior research evaluated incarceration history and cholesterol and blood glucose tests in young adults aged 29 years^16^; this study extends previous research by examining a wider range of preventive services use among older adults aged 40 to 65 years, an age range when risks of health problems are higher and people are eligible for preventive services, such as breast and colorectal cancer screenings. Associations of incarceration history and preventive services were attenuated after adjusting for educational attainment and health insurance coverage in multivariable models, suggesting that improving access to education and health insurance coverage among people with incarceration history might be associated with improved access to care. However, incarceration at a young age might interrupt education.^25^ Even after release from jail or prison, individuals might face challenges and discrimination at school, which can have lasting negative associations with employment opportunities and change life trajectories. Therefore, expanding programs to provide access to education and training in correctional facilities and educational supports after release will be needed. Meanwhile, our findings also suggested that improving health insurance coverage among people with incarceration history might improve access to care. Previous studies have found that incarceration history was associated with higher prevalence of uninsurance.^9,10,11^ Therefore, efforts to improve health insurance coverage among people with an incarceration history, such as presumptive Medicaid eligibility and provision of transitional care during reentry^33^ and federal policies to allow states to provide Medicaid coverage preceding the date of release,^34^ are warranted. Exposure to racism is associated with higher risk of incarceration and worse access to care among racialized populations^1,2,28^; thus, improving access to education and health insurance coverage among racialized populations is important for reducing disparities in health outcomes. Future research examining the interplay of racism, socioeconomic status, incarceration, access to care, and health outcomes is warranted.

In this study, we found that even after adjusting for demographic factors, educational attainment, and health insurance coverage, incarceration history was associated with lower chances of having a usual source of care, blood test for cholesterol level, and routine dental check up. Therefore, simply improving health insurance coverage might not be sufficient, and more comprehensive interventions might be needed to improve access to care among people with a history of incarceration.^35^ One approach is to link correctional facilities and community health systems to facilitate access to resources and a smooth reintegration.^35^ Providing training to health professionals in correctional facilities and the community could also be associated with improved cultural competence and the ability of health care professionals to demonstrate trustworthiness when caring for justice system–involved people.^35^ Meanwhile, programs to improve health literacy for incarcerated people or people with an incarceration history and help them navigate resources they can use when seeking care may also improve their access to care.

Our findings that incarceration was associated with lower chances of receiving colorectal and breast cancer screenings highlight cancer disparities. Cancer is a leading cause of disease-related death among justice system–involved people^36,37^ and will grow in importance as the justice system–involved populations are aging and cancer risk increases with age. Previous studies showed that incarceration was associated with higher cancer incidence, prevalence, and mortality rates.^38,39,40^ For example, a study using nationally representative data found that people with an incarceration history had higher age-adjusted prevalence of lung, cervical, and alcohol-related cancers compared with people without incarceration history.^38^ Using data from a few local jails or 1 state prison, earlier studies found that incarcerated people had lower rates of receiving colorectal or breast cancer screenings compared with the US average rates.^18,19,20,21,24^ In this study, we did not find significant differences in cervical cancer screening by incarceration history, which might be due to lack of accurate guideline-concordant screening information and relatively small sample sizes.

### Limitations

This study had several limitations. First, incarceration history was self-reported and subject to recall and social desirability bias. However, studies have shown that self-reported measures of interactions with the judicial system are consistent with official records.^41^ In addition, incarceration history was measured by a survey question about incarceration history at survey round 2 and about residing in jail, prison, or other detention facilities at the time of survey in later rounds. We were not able to identify people who had been incarcerated since round 3 but who did not reside in jail, prison, or any detention facilities at the time of the survey; therefore, we might underestimate the prevalence of incarceration history, and the associations of incarceration history and study outcomes may be biased toward null. In addition, we were not able to separately examine differential effects of jail or prison, which could be an important area for future studies. We were not able to examine potential contributions of income to the associations of incarceration history and access to and receipt of health care due to the high missing rate (17.2%). Although we used longitudinal data and measures of incarceration history before evaluation of study outcomes, our findings do not conclude causation as there might be other confounders, such as adverse childhood events, not included in the study due to lack of data availability. In addition, we were not able to control for the potential effects of Medicaid expansion within the Affordable Care Act because state of residence was not available from these data. Finally, we were not able to analyze receipt of up-to-date colorectal and cervical cancer screenings because we did not have complete information on the specific modalities (eg, timing of colonoscopy, Papanicolaou testing) of these measures.

## Conclusions

In this survey study using US national longitudinal data, the findings suggested that incarceration history was associated with worse access to and receipt of health care. The associations were attenuated after adjusting for educational attainment and health insurance coverage, suggesting that improving access to education at a young age and health insurance coverage might mitigate the disparities in access to care among people with an incarceration history.

## Notes

Supplement 1. eTable 1. Measures and exact wording of questions from the National Longitudinal Survey of Youth eTable 2. Rounds used for the analysis eTable 3. Differences in predicted margins in access to and receipt of care comparing people with and without incarceration history

Supplement 2. Data sharing statement
