# Supplementary material for: Incarceration History and Access to and Receipt of Health Care in the US
Source: JAMA Health Forum. 2024 Feb 23;5(2):e235318. doi: 10.1001/jamahealthforum.2023.5318 (PMC10891474; doi:10.1001/jamahealthforum.2023.5318)
Supplement: Supplement 1. — eTable 1. Measures and exact wording of questions from the National Longitudinal Survey of Youth eTable 2. Rounds used for the analysis eTable 3. Differences in predicted margins in access to and receipt of care comparing people with and without incarceration history [file jamahealthforum-e235318-s001.pdf]

Supplemental Online Content

Zhao J, Star J, Han X, et al. Incarceration history and access to and receipt of health care in the US. *JAMA Health Forum*. 2024;5(2):e235318. doi:10.1001/jamahealthforum.2023.5318

eTable 1. Measures and exact wording of questions from the National Longitudinal Survey of Youth

eTable 2. Rounds used for the analysis

eTable 3. Differences in predicted margins in access to and receipt of care comparing people with and without incarceration history

This supplemental material has been provided by the authors to give readers additional information about their work.

© 2024 Zhao J et al. *JAMA Health Forum.*

eTable 1. Measures and Exact Wording of Questions from the National Longitudinal Survey of Youth.

**Measures**	**Questions**	**Time frame in this study**	**Eligible population**	**Rounds included for the analysis**
Access to care	Do you have a health care provider that you can see when you are sick or need advice about your health?	At the time of the survey	All men and women	23-28
Preventive Services				
Physical exam	About how long has it been since your last general physical exam or routine checkup by a medical doctor or other health professional? Do not include a visit about a specific problem. Has it been…<br>- Never<br>- A year ago or less<br>- More than 1 year but not more than 2 years<br>- More than 2 years but not more than 3 years<br>- More than 3 years but not more than 5 years<br>- Over 5 years ago	2 years	All men and women	23-28
Flu shot	During the past 24 months, have you had any of the following medical tests or procedures? -- A flu shot?	2 years	All men and women	23-28
Blood pressure measured	During the past 24 months, have you had any of the following medical tests or procedures? -Have you had your blood pressure measured?	2 years	All men and women	23-28
Blood test for cholesterol	During the past 24 months, have you had any of the following medical tests or procedures? --A blood test for cholesterol?	2 years	All men and women	23-28
Blood test for diabetes or blood sugar levels	During the past 24 months, have you had any of the following medical tests or procedures? --A blood test for diabetes or blood sugar levels?	2 years	All men and women	23-28
Talk to dentist for routine check-up	During the past 24 months, have you seen or talked to any of the following types of doctors? --A dentist for a routine check-up or exam?	2 years	All men and women	23-28
Colorectal cancer screening	During the past 24 months, have you had any of the following medical tests or procedures?	2, 4, 6, 8, and	Men and women aged 50-75 years	23-28 for 2-year screening,

© 2024 Zhao J et al. *JAMA Health Forum.*

	--A colonoscopy or other test to screen for colorectal cancer?	10 years		24-28 for 4-year screening, 25-28 for 6-year screening, 26-28 for 8-year screening, and 27-28 for 10-year screening
Breast cancer screening	During the past 24 months, have you had any of the following medical tests or procedures?<br>--A mammogram or x-ray of the breast to search for cancer?	2 years	Women aged 50-74 years	23-28
Cervical cancer screening	During the past 24 months, have you had any of the following medical tests or procedures?<br>--A PAP smear?	2 and 4 years	Women aged 21-65 years who did not have a hysterectomy	23-28 for 2-year screening, 24-28 for 4-year screening

© 2024 Zhao J et al. *JAMA Health Forum.*

eTable 2. Rounds used for the analysis.

		Rounds at which incarceration questions were used to determine incarceration history (ever incarcerated)					
**Rounds at which access to and receipt of care questions were asked.**		2-22	23	24	25	26	27
Have health care provider to see when sick or need health advice	23	→					
	24	→	→				
	25	→	→	→			
	26	→	→	→	→		
	27	→	→	→	→	→	
	28	→	→	→	→	→	→
Receipt of preventive services including physical exam, flu shot, blood pressure check, cholesterol check, diabetes or blood sugar level check, and routine dental check-up, and breast cancer screening (women 50-74 years)	23	→					
	24	→	→				
	25	→	→	→			
	26	→	→	→	→		
	27	→	→	→	→	→	
	28	→	→	→	→	→	→
Colonoscopy or other colorectal cancer screening (among people aged 50-75 years)							
past 2 years	23	→					
	24	→	→				
	25	→	→	→			
	26	→	→	→	→		
	27	→	→	→	→	→	
	28	→	→	→	→	→	→
past 4 years	24	→	→				
	25	→	→	→			
	26	→	→	→	→		
	27	→	→	→	→	→	
	28	→	→	→	→	→	→
past 6 years	25	→	→	→			
	26	→	→	→	→		
	27	→	→	→	→	→	
	28	→	→	→	→	→	→
past 8 years	26	→	→	→	→		
	27	→	→	→	→	→	
	28	→	→	→	→	→	→
past 10 years	27	→	→	→	→	→	
	28	→	→	→	→	→	→
Pap smear for cervical cancer screening (among women aged 21-65 years)							
past 2 years	23	→					
	24	→	→				
	25	→	→	→			

© 2024 Zhao J et al. *JAMA Health Forum.*

	26						
	27						
	28						
past 4 years	24						
	25						
	26						
	27						
	28						

© 2024 Zhao J et al. *JAMA Health Forum.*

eTable 3. Differences in predicted margins in access to and receipt of care comparing people with and without incarceration history.

	With incarceration history		Without incarceration history		Difference	
	%	95% CI	%	95% CI	%	95% CI
Unadjusted models						
Have health care provider to see when sick or need health advice	68.00	65.07 to 70.94	87.68	87.09 to 88.28	-19.68	-22.67 to -16.69
Receipt of preventive services						
Physical exam	68.84	66.12 to 71.55	75.01	74.29 to 75.73	-6.18	-8.99 to -3.36
Flu shot	37.08	33.78 to 40.38	43.03	42.04 to 44.01	-5.95	-9.4 to -2.51
Blood pressure measured	83.11	80.86 to 85.35	91.33	90.75 to 91.91	-8.22	-10.54 to -5.91
Blood test for cholesterol	58.07	54.92 to 61.23	71.64	70.84 to 72.44	-13.57	-16.82 to -10.32
Blood test for diabetes or blood sugar levels	60.23	57.2 to 63.25	66.80	65.97 to 67.64	-6.58	-9.72 to -3.44
Talk to dentist for routine check-up	45.81	42.89 to 48.73	69.05	68.17 to 69.93	-23.24	-26.29 to -20.19
Colonoscopy or other colorectal cancer screening						
past 2 years	31.98	28.81 to 35.14	32.60	31.84 to 33.35	-0.62	-3.88 to 2.63
past 4 years	46.11	42.24 to 49.98	46.16	45.2 to 47.11	-0.05	-4.03 to 3.94
past 6 years	55.25	51.03 to 59.46	54.44	53.36 to 55.52	0.81	-3.55 to 5.16
past 8 years	62.20	57.82 to 66.57	61.10	59.89 to 62.32	1.09	-3.45 to 5.63
past 10 years	68.37	63.91 to 72.83	68.43	67.14 to 69.73	-0.07	-4.71 to 4.58
Mammogram or x-ray for breast cancer screening						
past 2 years	60.32	49.5 to 71.14	72.90	71.75 to 74.04	-12.58	-23.46 to -1.7
Pap smear for cervical cancer screening						
past 2 years	61.97	52.34 to 71.6	67.92	66.84 to 68.99	-5.95	-15.64 to 3.75
past 4 years	74.63	65.02 to 84.24	81.01	79.98 to 82.04	-6.37	-16.04 to 3.29
Model 1 (models adjust for age, sex, race and ethnicity, parents' highest educational attainment, rural urban status (last round), region (last round), number of conditions (last round)), and survey year.						
Have health care provider to see when sick or need health advice	72.12	68.57 to 75.66	87.41	85.18 to 89.64	-15.30	-18.27 to -12.33
Receipt of preventive services						
Physical exam	69.61	65.99 to 73.22	74.16	71.47 to 76.84	-4.55	-7.28 to -1.82
Flu shot	43.21	38.57 to 47.85	46.33	42.82 to 49.85	-3.12	-6.64 to 0.4
Blood pressure measured	85.61	82.71 to 88.51	91.61	89.61 to 93.62	-6.00	-8.35 to -3.66
Blood test for cholesterol	59.46	55.3 to 63.63	72.20	69.12 to 75.29	-12.74	-15.94 to -9.54
Blood test for diabetes or blood sugar levels	61.38	57.22 to 65.54	69.37	66.26 to 72.49	-7.99	-11.12 to -4.87
Talk to dentist for routine check-up	51.06	46.94 to 55.17	66.02	63.09 to 68.95	-14.96	-18.21 to -11.71
Colonoscopy or other colorectal cancer screening						
past 2 years	28.65	24.81 to 32.49	30.76	28.16 to 33.36	-2.11	-5.3 to 1.08
past 4 years	42.59	37.56 to 47.61	45.27	41.68 to 48.86	-2.68	-6.64 to 1.28
past 6 years	50.76	45.03 to 56.49	54.25	49.93 to 58.56	-3.49	-7.79 to 0.82
past 8 years	58.55	52.26 to 64.84	62.87	57.99 to 67.76	-4.33	-8.86 to 0.21

© 2024 Zhao J et al. *JAMA Health Forum.*

past 10 years	65.63	59 to 72.26	70.34	65.1 to 75.58	-4.71	-9.41 to -0.01
Mammogram or x-ray for breast cancer screening						
past 2 years	54.98	43.86 to 66.1	68.23	63.61 to 72.84	-13.24	-23.8 to -2.69
Pap smear for cervical cancer screening						
past 2 years	57.34	46.75 to 67.94	61.87	57.15 to 66.59	-4.52	-14.48 to 5.44
past 4 years	71.76	61.53 to 81.99	76.84	72.57 to 81.1	-5.08	-14.87 to 4.72
Model 2 (models adjust for age, sex, race and ethnicity, parents' highest educational attainment, rural urban status (last round), region (last round), number of conditions (last round),survey year, and educational attainment (last round))						
Have health care provider to see when sick or need health advice	72.14	68.67 to 75.62	84.68	82.46 to 86.91	-12.54	-15.48 to -9.6
Receipt of preventive services						
Physical exam	69.56	65.94 to 73.18	72.70	69.97 to 75.42	-3.14	-5.88 to -0.4
Flu shot	43.75	39.11 to 48.39	44.96	41.42 to 48.5	-1.21	-4.74 to 2.33
Blood pressure measured	85.54	82.65 to 88.43	89.73	87.64 to 91.83	-4.20	-6.57 to -1.82
Blood test for cholesterol	60.08	55.93 to 64.22	70.05	66.91 to 73.19	-9.97	-13.13 to -6.81
Blood test for diabetes or blood sugar levels	61.92	57.76 to 66.08	67.66	64.47 to 70.85	-5.74	-8.86 to -2.63
Talk to dentist for routine check-up	52.35	48.18 to 56.52	62.98	59.94 to 66.02	-10.64	-13.9 to -7.37
Colonoscopy or other colorectal cancer screening						
past 2 years	28.68	24.84 to 32.52	29.98	27.34 to 32.62	-1.30	-4.51 to 1.91
past 4 years	54.66	49.68 to 59.64	56.17	52.63 to 59.71	-1.51	-5.48 to 2.47
past 6 years	60.99	55.32 to 66.65	62.81	58.57 to 67.04	-1.82	-6.13 to 2.49
past 8 years	66.79	60.61 to 72.97	69.34	64.56 to 74.11	-2.55	-7.07 to 1.97
past 10 years	72.16	65.64 to 78.69	75.39	70.23 to 80.55	-3.23	-7.94 to 1.48
Mammogram or x-ray for breast cancer screening						
past 2 years	54.78	43.9 to 65.65	66.05	61.34 to 70.76	-11.27	-21.56 to -0.99
Pap smear for cervical cancer screening						
past 2 years	57.75	47.31 to 68.18	60.40	55.65 to 65.14	-2.65	-12.47 to 7.17
past 4 years	76.30	66.26 to 86.34	79.64	75.48 to 83.81	-3.35	-13.01 to 6.32
Model 3 (models adjust for age, sex, race and ethnicity, parents' highest educational attainment, rural urban status (last round), region (last round), number of conditions (last round), survey year, and health insurance status (last round))						
Have health care provider to see when sick or need health advice	69.38	62.85 to 75.91	77.54	71.33 to 83.74	-8.16	-10.52 to -5.79
Receipt of preventive services						
Physical exam	71.98	65.59 to 78.38	71.51	65.48 to 77.53	0.48	-2.06 to 3.01
Flu shot	43.09	35.41 to 50.76	42.36	35.29 to 49.42	0.73	-2.69 to 4.16
Blood pressure measured	85.26	80.36 to 90.16	88.07	83.6 to 92.54	-2.81	-5.05 to -0.57
Blood test for cholesterol	55.56	46.87 to 64.25	62.53	54.18 to 70.88	-6.97	-9.91 to -4.03
Blood test for diabetes or blood sugar levels	63.52	57.52 to 69.52	66.76	61.32 to 72.21	-3.24	-6.18 to -0.3
Talk to dentist for routine check-up	48.68	40.31 to 57.05	57.20	49.27 to 65.12	-8.52	-11.53 to -5.5
Colonoscopy or other colorectal cancer screening						
past 2 years	26.62	16.38 to 36.86	25.51	15.63 to 35.39	1.11	-2.01 to 4.24
past 4 years	48.28	36.66 to 59.89	46.29	35.23 to 57.36	1.98	-1.87 to 5.83
past 6 years	54.76	44.92 to 64.59	53.51	44.45 to 62.57	1.25	-2.92 to 5.41

© 2024 Zhao J et al. *JAMA Health Forum.*

past 8 years	66.82	60.15 to 73.49	66.58	61.27 to 71.89	0.24	-4.15 to 4.62
past 10 years	70.84	63.68 to 78	71.07	65.27 to 76.88	-0.24	-4.91 to 4.43
Mammogram or x-ray for breast cancer screening						
past 2 years	59.98	49.75 to 70.22	69.49	64.8 to 74.17	-9.50	-18.96 to -0.05
Pap smear for cervical cancer screening						
past 2 years	55.03	36.23 to 73.83	56.62	40.28 to 72.95	-1.59	-11.28 to 8.1
past 4 years	76.08	65.86 to 86.3	78.53	73.97 to 83.08	-2.45	-12.09 to 7.2
Model 4 (models adjust for age, sex, race and ethnicity, parents' highest educational attainment, rural urban status (last round), region (last round), number of conditions (last round), survey year, educational attainment (last round), and health insurance status (last round))						
Have health care provider to see when sick or need health advice	69.62	63.15 to 76.1	76.28	70.13 to 82.43	-6.66	-9.02 to -4.3
Receipt of preventive services						
Physical exam	72.05	65.59 to 78.52	70.98	64.87 to 77.1	1.07	-1.48 to 3.61
Flu shot	43.99	36.38 to 51.61	42.11	35.11 to 49.1	1.88	-1.56 to 5.33
Blood pressure measured	85.43	80.51 to 90.35	87.00	82.48 to 91.53	-1.58	-3.83 to 0.68
Blood test for cholesterol	56.55	47.9 to 65.19	61.87	53.55 to 70.18	-5.32	-8.25 to -2.39
Blood test for diabetes or blood sugar levels	64.36	58.46 to 70.27	66.27	60.91 to 71.62	-1.91	-4.85 to 1.04
Talk to dentist for routine check-up	50.28	41.63 to 58.92	55.89	47.65 to 64.12	-5.61	-8.66 to -2.56
Colonoscopy or other colorectal cancer screening						
past 2 years	26.62	16.34 to 36.89	25.18	15.27 to 35.1	1.44	-1.7 to 4.57
past 4 years	58.62	46.53 to 70.71	56.31	44.75 to 67.87	2.31	-1.57 to 6.18
past 6 years	62.46	51.31 to 73.61	60.50	50.02 to 70.98	1.96	-2.22 to 6.14
past 8 years	70.45	61.89 to 79	69.37	61.81 to 76.94	1.08	-3.31 to 5.46
past 10 years	75.48	66 to 84.96	75.07	66.54 to 83.61	0.41	-4.27 to 5.08
Mammogram or x-ray for breast cancer screening						
past 2 years	59.17	49.09 to 69.26	67.38	62.63 to 72.12	-8.21	-17.51 to 1.1
Pap smear for cervical cancer screening						
past 2 years	55.00	36.65 to 73.36	55.35	39.44 to 71.26	-0.34	-9.95 to 9.26
past 4 years	78.64	68.45 to 88.83	79.73	74.94 to 84.52	-1.09	-10.62 to 8.44

© 2024 Zhao J et al. *JAMA Health Forum.*
