# Supplementary material for: Incarceration History and Access to and Receipt of Health Care in the US
Source: JAMA Health Forum. 2024 Feb 23;5(2):e235318. doi: 10.1001/jamahealthforum.2023.5318 (PMC10891474; doi:10.1001/jamahealthforum.2023.5318)
Supplement: Supplement 2. — Data sharing statement [file jamahealthforum-e235318-s002.pdf]

Data Sharing Statement

Zhao. Incarceration History and Access to and Receipt of Health Care in the US. *JAMA Health Forum*. Published February 23, 2024. doi:10.1001/jamahealthforum.2023.5318

Data
Data available: No

Additional Information
Explanation for why data not available: The data is publicly available and researchers can get the data after creating an account and signing the DUA.
